# Development of monoclonal antibodies and quantitative ELISAs targeting insulin-degrading enzyme

**DOI:** 10.1186/1750-1326-4-39

**Published:** 2009-10-16

**Authors:** Anthony DelleDonne, Naomi Kouri, Lael Reinstatler, Tomoko Sahara, Lilin Li, Ji Zhao, Dennis W Dickson, Nilufer Ertekin-Taner, Malcolm A Leissring

**Affiliations:** 1 Department of Neuroscience, Mayo Clinic, 4500 San Pablo Road S, Jacksonville, FL 32224, USA; 2 Department of Molecular Therapeutics, The Scripps Research Institute, Scripps Florida, 5353 Parkside Dr, Jupiter, FL 32458, USA; 3 Department of Neurology, Mayo Clinic College of Medicine, 4500 San Pablo Road S, Jacksonville, FL 32224, USA

## Abstract

**Background:**

Insulin-degrading enzyme (IDE) is a widely studied zinc-metalloprotease implicated in the pathogenesis of type 2 diabetes mellitus, Alzheimer disease (AD) and varicella zoster virus infection. Despite more than six decades of research on IDE, progress has been hampered by the lack of well-characterized reagents targeting this biomedically important protease. To address this important need, we generated and characterized new mouse monoclonal antibodies (mAbs) targeting natively folded human and rodent IDE.

**Results:**

Eight monoclonal hybridoma cell lines were derived in house from mice immunized with full-length, natively folded, recombinant human IDE. The mAbs derived from these lines were shown to detect IDE selectively and sensitively by a wide range of methods. Two mAbs in particular—designated 6A1 and 6H9—proved especially selective for IDE in immunocytochemical and immunohistochemical applications. Using a variety of methods, we show that 6A1 selectively detects both human and rodent IDE, while 6H9 selectively detects human, but not rodent, IDE, with both mAbs showing essentially no cross reactivity with other proteins in these applications. Using these novel anti-IDE mAbs, we also developed sensitive and quantitative sandwich ELISAs capable of quantifying IDE levels present in human brain extracts.

**Conclusion:**

We succeeded in developing novel mAbs that selectively detect rodent and/or human IDE, which we have shown to be suitable for a wide range of applications, including western blotting, immunoprecipitation, immunocytochemistry, immunohistochemistry, and quantitative sandwich ELISAs. These novel anti-IDE mAbs and the assays derived from them constitute important new tools for addressing many unresolved questions about the basic biology of IDE and its role in multiple highly prevalent human diseases.

## Background

Insulin-degrading enzyme (IDE; EC 3.4.24.56; a.k.a. insulysin, insulinase, insulin protease) is an atypical zinc-metalloprotease that hydrolyzes several biomedically important intermediate-sized peptide substrates, including insulin, insulin-like growth factor-2, glucagon, amylin, and the amyloid β-protein [1-3]. IDE is implicated in the pathogenesis of Alzheimer disease (AD) [4,5] and type-2 diabetes mellitus [6-8], and has also been identified as the cellular receptor for varicella zoster virus infection and cell-to-cell spread [9].

Despite the clear biomedical significance of this protease, many fundamental questions about the basic biology of IDE remain unresolved, due in part to a lack of sufficiently selective reagents targeting this ubiquitous protease. In particular, the precise subcellular localization of IDE remains poorly defined. Although IDE is well-established to reside in cytosol [1] and mitochondria [10], reports of IDE's localization to other pathophysiologically important subcellular compartments—such as endosomes [11]—have not been confirmed by microscopic analysis of intact cells with well-characterized anti-IDE antibodies. Moreover, the mechanisms underlying the export of IDE from the cell are completely unknown, though it has recently been demonstrated that they involve an unconventional, non-classical secretion pathway [12]. Methods capable of detecting and quantifying secreted forms of IDE would greatly facilitate the elucidation of this important pathway. Finally, it will be important to detect genetically or environmentally induced variations in IDE protein levels, which will require the development of assays permitting accurate quantification of IDE levels in relevant tissues.

To help close these gaps in our understanding of the biology of IDE, we developed eight novel mouse mAbs that detect rodent and/or human IDE in diverse applications in a highly selective and species-specific manner. Notably, a subset of these mAbs were particularly well suited for detecting endogenous IDE by immunocytochemistry and immunohistochemistry. We also describe the development of sensitive and quantitative sandwich ELISAs capable of detecting variations in IDE levels in human brain extracts. Collectively, these novel anti-IDE mAbs, and the ELISA incorporating them, constitute important new tools for investigating both the basic biology of IDE and its potential derangement in disease.

## Results

Detailed methods for all experimental procedures are provided in the Additional File 1.

### Generation of Monoclonal Hybridomas Expressing Anti-IDE mAbs

To generate mAbs targeting IDE, BALB/ByJ mice were immunized in house with highly purified, full-length, natively folded, recombinant human IDE. Spleen cells were harvested and fused with SP2/0-Ag14 myeloma cells, and monoclonal hybridomas were selected for by growth in HAT medium. From among a total of 576 hybridoma lines, 8 clones were selected and expanded based on their reactivity against a second, natively folded, recombinant human IDE protein. The anti-IDE mAbs derived from these hybridomas were purified by protein G-sepharose chromatography, and their isotypes and half-titers were determined (Table 1).

**Table 1:** Properties of anti-IDE monoclonal antibodies

**Clone:**	**2A1**	**3D8**	**4B4**	**4C5**	**4H5**	**4H7**	**6A1**	**6H9**
Isotype:	IgG_1 _κ	IgG_1 _κ	IgG_1 _κ	IgG_2a _κ	IgG_2a _κ	IgG_1 _κ	IgG_2b _κ	IgG_2a _κ

ELISA half-titer (ng/mL):	250	32	32	8	4	125	2	8

### Western Blotting and Immunoprecipitation

**Figure 1:**
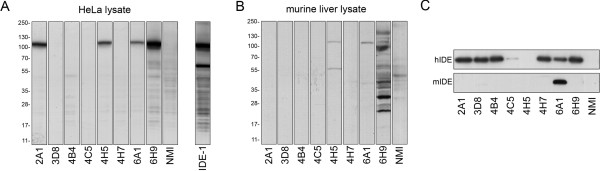
**Detection of human and rodent IDE by 6A1 and 6H9 using western blotting and immunoprecipitation**. ***A***, Western blots of HeLa cell lysate (30 μg/lane) detected with individual anti-IDE mAbs (10 μg/mL) or, as a control, equivalent amounts of normal mouse IgG (NMI). For comparative purposes, the same cell lysate was probed with αIDE-1, a well-characterized rabbit polyclonal anti-IDE antibody ([13]; generous gift of D. Selkoe, Harvard Medical School). ***B***, Western blots of mouse liver extracts (30 μg/lane) detected with anti-IDE mAbs or NMI (10 μg/mL). Note that 6H9 labels multiple non-specific bands, but does not label rodent IDE *per se *(see text). ***C***, Immunoprecipitation of human (*upper panel*) or rodent (*lower panel*) IDE by anti-IDE mAbs or NMI and detected by western blotting with αIDE-1. Note that rodent IDE was successfully immunoprecipitated by 6A1, exclusively.

By western blotting, endogenous human IDE present in HeLa cell extracts was readily detected by 4 anti-IDE mAbs: 2A1, 4H5, 6A1 and 6H9 (Fig. 1A). These mAbs each detected a prominent ~110-kDa band identical in size to that detected by the well characterized rabbit polyclonal antibody, αIDE-1 [13] (Fig. 1A). Little to no non-specific staining was observed, which in no case exceeded that detected by identical amounts of normal mouse IgG (Fig. 1A). Rodent IDE extracted from mouse liver, by contrast, was detected by 4H5 and 6A1, but not by other mAbs (Fig. 1B). 6H9 detected multiple bands, including some near ~110 kDa (Fig. 1B); however, these bands were found to be non-specific, because an identical pattern was observed in cell extracts derived from wild-type and IDE-KO mice (not shown).

With the exception of 4H5, human IDE was successfully immunoprecipitated by all anti-IDE mAbs, albeit the efficiency of 4C5 was less than the other mAbs (Fig. 1C). By contrast, rodent IDE was successfully immunoprecipitated by only a single mAb, 6A1 (Fig. 1C).

### Immunocytochemistry

**Figure 2:**
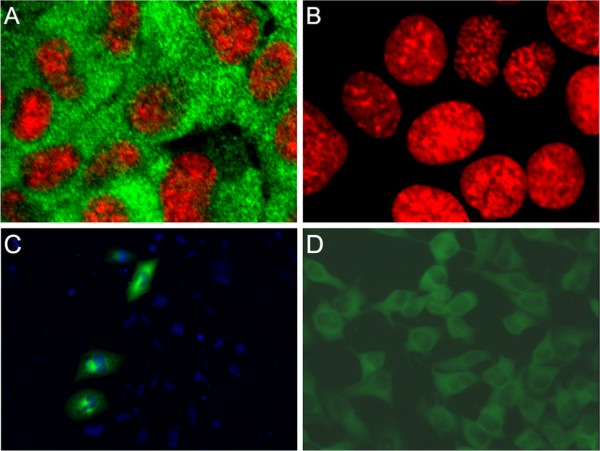
**Immunocytochemistry using IDE antibodies 6A1 and 6H9**. ***A, B***, Immortalized hepatocytes from wild-type (***A***) and IDE-KO (***B***) mice immunolabeled with 6A1 (*green*, 10 μg/mL) and visualized under identical conditions by laser confocal microscopy (100× magnification). Nuclei are stained with propidium iodide (*red*). Note the complete absence of 6A1 immunoreactivity in IDE-KO cells (***B***). ***C***, CHO cells transiently transfected with human IDE cDNA, labeled with 6H9 (*green*, 20 μg/mL). Nuclei are stained with DAPI (*blue*). Note absence of 6H9 immunoreactivity in non-transfected cells. ***D***, HeLa cells stained with 6H9 (*green*, 10 μg/mL), showing that this mAb can detect endogenous levels of human IDE. Images in ***C ***and ***D ***were acquired using conventional fluorescent microscopy (20× magnification).

To evaluate the specificity of our anti-IDE mAbs for detecting rodent IDE by immunocytochemistry, we compared the signal detected in immortalized hepatocytes derived from wild-type mice or mice lacking IDE (IDE-KO mice). From among all antibodies tested, including multiple commercially available ones, 6A1 was unique in strongly labeling wild-type hepatocytes (Fig. 2A), while showing no immunoreactivity in IDE-KO hepatocytes imaged under identical conditions (Fig. 2B). Consistent with previous results [14], IDE was found to be widely distributed throughout the cytoplasm and largely excluded from the nucleus by high-resolution confocal microscopy (Fig. 2A).

To evaluate the specificity of our mAbs for detecting human but not rodent IDE by immunocytochemistry, we analyzed Chinese hamster ovary (CHO) cells transiently transfected with a vector encoding human IDE. Among the antibodies tested, superior results were obtained with 6H9, which was found to intensely stain cells expressing human IDE, while showing no background staining in neighboring, nontransfected cells expressing rodent IDE (Fig. 1C). These results are consistent with the western blotting and immunoprecipitation results obtained for this mAb (Fig 1). 6H9 also readily detected endogenous levels of human IDE, as revealed by prominent staining present in unmodified HeLa cells (Fig. 1D).

### Immunofluorescence and Immunohistochemistry

**Figure 3:**
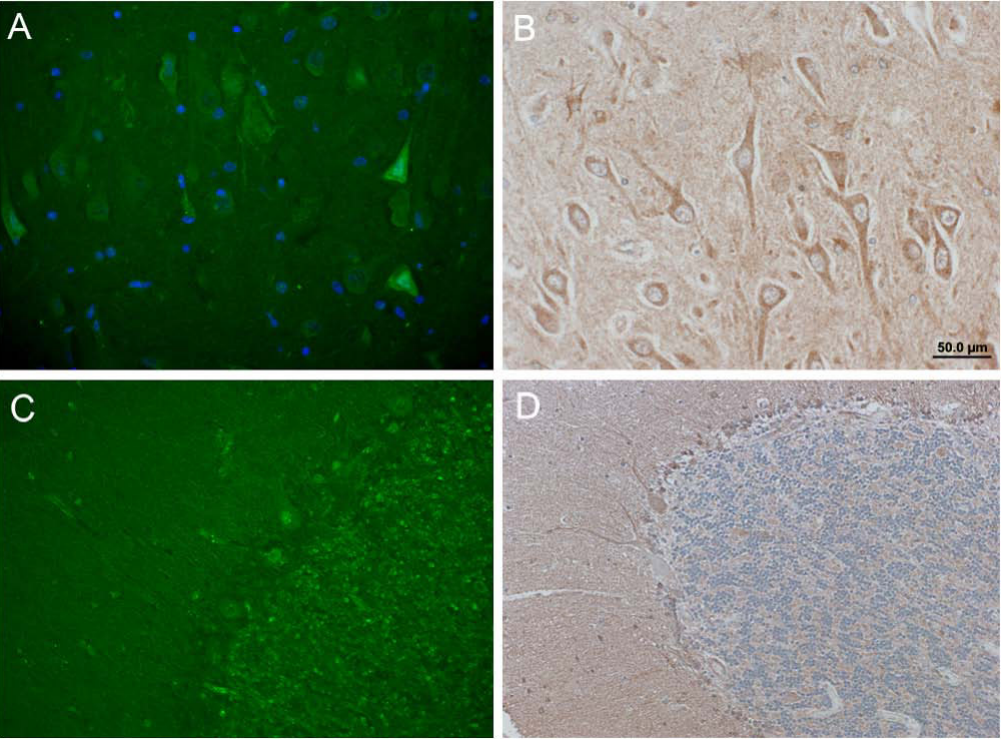
**Immunofluorescent and immunohistochemical detection of IDE in normal human brain tissue**. ***A, B***, Hippocampal sections of paraffin-embedded human brain tissue stained with 6H9 (100 μg/mL) and detected by immunofluorescence (***A***) and immunohistochemistry (***B***). Note the presence of IDE in pyramidal neurons and glia detected by both methods. ***C, D***, Cerebellar sections stained with 6H9 (100 μg/mL) and detected by immunofluorescence (***C***) and immunohistochemistry (***D***). Note the presence of IDE in the cell bodies of Purkinje cells.

The suitability of the anti-IDE mAbs for immunohistochemical applications was assessed by staining paraffin-embedded sections from pathologically unaffected human hippocampus and cerebellum. 6H9 (Fig. 3) and 6A1 (not shown) showed highly similar patterns of staining relative to one another as determined by both immunofluorescent (Fig. 3A, C) and immunohistochemical (Fig. 3B, C) methods. As expected from the ubiquitous expression of IDE, immunoreactivity was broadly distributed, though some cell-type specific variations in staining intensity were apparent. Notably, a subset of neuronal cells showed comparatively higher IDE immunoreactivity, including CA1 pyramidal cells in the hippocampus (Fig. 3A, B) and Purkinje cells in the cerebellum (Fig. 3C, D).

### IDE Sandwich ELISAs

**Figure 4:**
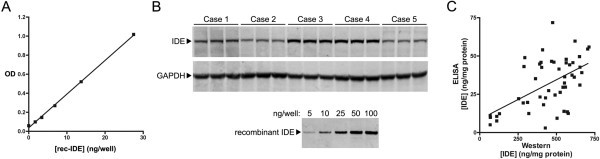
**Development of a sandwich ELISA for quantification of human IDE levels in brain extracts**. ***A***, Quantitative detection of recombinant human IDE by ELISA using 6H9 for capture and HRP-conjugated 6A1 for detection. ***B***, Representative western blot showing variations in IDE protein levels in human cerebellar extracts detected with 2A1. ***C***, Quantification of IDE by western blotting and 6H9/6A1 sandwich ELISA in 49 human cerebellar samples. For detailed methods, see Additional File 1.

We next sought to develop a sandwich ELISA capable of detecting and quantifying IDE present in human brain extracts. From among several configurations tested, we elected to characterize a sandwich ELISA using 6H9 for capture and horse radish peroxidase (HRP)-conjugated 6A1 for detection, as these two antibodies had the most robust combined results with the western blot, immunofluorescent and immunohistochemical assays. This ELISA showed a linear response to a wide range of concentrations of recombinant IDE, where the minimum amount of recombinant IDE that was repeatedly and reliably detected was 1.7 ng/well, or 156 pM in 100 μL (Fig. 4A).

To validate the ability of this ELISA to detect variations in IDE levels from human brain samples, we quantified IDE levels in cerebellar extracts from a large set of autopsied AD brains both by western blot analysis with 2A1 (see Fig. 4B) and by 6H9/6A1 sandwich ELISA. For both methods, absolute IDE levels were determined by calibration to internal recombinant human IDE standards. After appropriate quality control measures and normalization to internal control samples (see Additional File 1), IDE levels within a total of 49 human cerebellar samples were successfully measured by both methodologies (Fig. 4C). Despite the different set of antibodies employed and potential batch effects, we observed a highly significant correlation between results obtained with the two methodologies (p < 0.0001, r^2 ^= 0.3). Overall, the 6H9/6A1 ELISA consistently detected less IDE protein (7 ± 1%, mean ± SD) relative to that detected by western blotting (see *Discussion*). Using a subset of brain samples as a reference, qualitatively and quantitatively similar results were also obtained using ELISAs configured with multiple different anti-IDE mAbs combinations (not shown).

## Discussion

In the present study, we succeeded in developing 8 monoclonal hybridoma lines in house that express a versatile set of anti-IDE mAbs. Two mAbs in particular—6A1 and 6H9—were found to be useful in a wide array of applications, detecting human and rodent IDE in a highly selective and species-specific manner. 6A1 was found to detect both human and rodent IDE by western blotting, immunoprecipitation, immunocytochemistry, and immunohistochemistry. 6H9, by contrast, detected human but not rodent IDE, as determined by the same methods.

We also developed sandwich ELISAs capable of detecting human IDE in brain extracts and validated the ELISA results with quantitative western blot analysis. There was a significant correlation between IDE levels detected by western blotting with 2A1 and with the 6H9/6A1 sandwich ELISA. Despite the strong correlation between the two methods, the absolute amounts of IDE detected by ELISA were consistently lower than those detected by western blotting. This disparity may be attributable technical considerations, such as the particular protein extraction conditions used in this study (see Additional File 1), which could have denatured IDE sufficiently to affect its detection by ELISA. Future studies comparing the outcome obtained under different extraction conditions should resolve this question. On the other hand, several intriguing biological explanations also exist. For example, endogenous IDE might normally be complexed to other proteins, or may contain post-translational modifications, either or both of which could sterically block or remove the epitopes recognized by the antibodies used for ELISAs. Alternatively, or in addition, it may be that a substantial portion of IDE present in post-mortem extracts is itself not natively folded or is modified in other ways.

Although outside the scope of this methodology paper, it is notable that there was substantial variation in absolute IDE levels (~10-fold) detected by both ELISA and Western blotting in the large set of AD cerebellar samples we examined. In the future, it will be important to evaluate whether these changes correlate with genetic or other risk factors for AD. Further insight into these and many other important questions will be facilitated by the development of this well-characterized and versatile set of anti-IDE mAbs.

## Abbreviations

AD: Alzheimer disease; ELISA: enzyme-linked immunosorbent assay; HAT: hypoxanthine, aminopterin, and thymidine; HRP: horse radish peroxidase; IDE: insulin-degrading enzyme; IDE-KO: insulin-degrading enzyme knockout; mAb: monoclonal antibody.

## Competing interests

The authors declare that they have no competing interests.

## Authors' contributions

AD conducted immunohistochemical and immunofluorescent staining, assisted with microscopy and wrote the manuscript. NK conducted western blot analyses and ELISAs on human brain extracts. LR participated in fluorescence microscopy, generated HRP-conjugated IDE mAbs, and tested and optimized IDE ELISAs. JZ conducted fluorescence confocal microscopy. LL prepared and purified recombinant IDE proteins, immunized mice, harvested B cells, generated hybridomas, tested hybridoma clones for IDE immunoreactivity in various formats, expanded and isotyped selected clones, generated immortalized murine hepatocyte cell lines, and tested mAbs by western blotting and immunoprecipitation. TS conducted immunocytochemistry on transiently transfected CHO cells. DWD aided in antibody analysis and characterization on human tissue samples. NET designed experiments, analyzed data and wrote the manuscript. MAL designed experiments, participated in fluorescence microscopy, analyzed data and wrote the manuscript.

## Supplementary Material

Additional file 1 **Supplementary Experimental Methods**. Detailed experimental methods. Click here for file
