# Supplementary material for: Development of monoclonal antibodies and quantitative ELISAs targeting insulin-degrading enzyme
Source: Mol Neurodegener. 2009 Oct 16;4:39. doi: 10.1186/1750-1326-4-39 (PMC2768702; doi:10.1186/1750-1326-4-39)
Supplement: Additional file 1 — Supplementary Experimental Methods. Detailed experimental methods. [file 1750-1326-4-39-S1.DOC]

# Supplementary Experimental Methods

### Chemicals

Unless otherwise noted, all chemicals were of reagent quality and purchased from Sigma Aldrich.

### Expression and Purification of Recombinant IDE

Recombinant human IDE used for immunizations was produced in bacteria as an N-terminally tagged glutathione S-transferase (GST) fusion protein, purified on a glutathione-Sepharose column (GE Healthcare), then cleaved from the column-bound GST tag with PreScission protease as described previously [1]. Recombinant IDE for use in the screening of mAbs and the serum of immunized mice was generated and purified as a maltose-binding protein (MBP)-IDE fusion protein containing a hexahistadine tag attached to the N-terminus of MBP. Briefly, the open reading frame of human IDE (starting at amino acid 42) was cloned into pMCSG-V19-6xHis-MBP (generous gift of Dr. Kendall Nettles, Scripps Florida), and the resulting construct was used to transform the Rosetta strain of Escherichia coli (GE Healthcare). Protein expression was induced by treatment with 50 µM IPTG for 16 h at 27 ^o^C. Bacterial pellets were harvested at 4 ^o^C, resuspended in Buffer A (50 mM Tris-HCl, 300 mM NaCl, 5 mM ß-ME, 20 mM imidazole, 1 mM PMSF, adjusted to pH 8.0), lysed by sonication, and centrifuged at 100,000g to remove cellular debris. The resulting supernatant was applied to Ni-NTA Superflow beads (Qiagen), washed extensively with Buffer A without PMSF, and eluted with the latter buffer containing 250 mM imidazole. IDE was then separated from MBP by treatment with tobacco etch virus (TEV) protease, and applied to a 70-kDa molecular weight cutoff (MWCO) spin column (Amicon).

Additional purification of both recombinant IDE proteins was carried out by gel-filtration using a Superdex 200 column (GE Healthcare) essentially as described [2]. Peak fractions were pooled and dialyzed overnight against 2 changes of PBS supplemented with 1 mM DTT. The purified recombinant proteins were concentrated using 30-kDa MWCO spin columns to a final concentration of ~ 5 mg/mL, flash frozen in liquid N_2_, then stored at -80 ^o^C.

### Immunizations

Six-week-old female BALB/ByJ mice (Jackson Labs) were immunized by intraperitoneal injection with 50 µg recombinant human IDE reconstituted in the MPL+TDM Adjuvant System (Sigma Aldrich) according to manufacturer’s recommendations. Immunized mice were boosted an additional 6 times by the same treatment administered at ~3-week intervals, then a final time 3 days before the harvesting of spleen cells. The anti-IDE antibody titer within the plasma of immunized mice was monitored using the same methods used for the screening of hybridoma lines (see below).

### Cell Fusion and Selection of Hybridoma Lines

The SP2/0-Ag14 myeloma cell line (American Type Culture Collection) was cultured in serum-free Complete DMEM (*i.e.*, DMEM supplemented with 1 mM sodium pyruvate, 4 mM L-glutamine, 1% penicillin/streptomycin (Gibco BRL)). Two mice showing the highest serum anti-IDE antibody titer were euthanized by cervical dislocation, and spleen was dissected and transferred to serum-free Complete DMEM. Spleen cells were dissociated by mincing followed by successive passage through 25G and 18G needles, then the resulting suspension was strained through sterile cheese cloth to remove non-cellular debris. The dissociated spleen cells were combined with SP2/0-Ag14 myeloma cells at a 5:1 ratio, pelleted by centrifugation, then resuspended in 50% PEG 1500 (Roche) in serum-free Complete DMEM. The resulting cell suspension was distributed into 96-well cell culture plates (Corning) at 100 µL/well. One day after cell fusion, selection was initiated by addition of 100 µL/well DMEM supplemented with 1 mM sodium pyruvate and a 1X solution of hypoxanthine, aminopterin, and thymidine (HAT), and maintained by supplementation with fresh DMEM/pyruvate/HAT medium every 2 days. Two weeks after cell fusion, the medium was exchanged with DMEM supplemented with 50 mM HEPES, pH 7.4, 1 mM sodium pyruvate and 1X hypoxanthine and thymidine. Following selection of positive wells (see below), putative clones were subcloned an additional 2 times to ensure the isolation of true monoclonal hybridoma lines.

### ELISA-based Screening of Hybridoma Supernatant and Mouse Serum

96-well High Binding Microplates (Corning) were coated with 10 µg/mL recombinant human IDE (derived from the MBP-IDE fusion protein) diluted in TBS (50 mM Tris-HCl, 100 mM NaCl, pH 7.4). Plates were blocked by incubation with TBS supplemented with 1% BSA for 2 h at 22 ^o^C. Hybridoma supernatant or fresh serum from immunized mice was added to the plates (100 µL/well), then diluted 1:1 in TBS multiple times. Plates were washed 5 times with ELISA Wash Buffer (TBS supplemented with 0.2% Tween 20). HRP-conjugated goat anti-mouse IgG (H+L) antibody (Abcam) diluted 1:12,000 in TBS/1% BSA was then added and allowed to react for 30 min at 37 ^o^C. Following 6 washes with ELISA Wash Buffer, peroxidase activity was quantified by using the Enzolyte MG Phosphate Assay Kit (Anaspec) according to manufacturer’s recommendations.

### Purification and Isotyping of Monoclonal Anti-IDE Antibodies

Hybridoma lines were expanded in RPMI 1640 medium (Gibco BRL) supplemented with 1X Hybridoma Fusion and Cloning Supplement (Roche) in 2-L LAMPIRE™ Cell Culture Bags (Lampire Biological Laboratories), and the resulting mAbs were purified on a Protein G Sepharose 4 Fast Flow column (GE Healthcare) according to manufacturer’s recommendations. The purified antibodies were exchanged into PBS and concentrated to 1 – 2 mg/mL using 70-kDa MWCO spin columns (Amicon), and stored frozen in aliquots at -80 ^o^C. Antibodies were isotyped using the IsoStrip Mouse Monoclonal Antibody Isotyping Kit (Roche) according to manufacturer’s recommendations.

### Cell Culture and Transfections

SV40-transformed wild-type and IDE-KO murine hepatocytes were generated and maintained as described previously [3]. HeLa cells [3] and CHO cells [4] were maintained as described previously. CHO cells were transfected with a mammalian expression construct expressing human IDE as described [4].

### Extraction of Protein from Cells, Mouse Liver, and Human Brain Samples

Protein extracts containing endogenous human and rodent IDE were obtained from HeLa cells and wild-type C57Bl/6 mouse liver, respectively. Briefly, confluent monolayers of HeLa cells were washed 2 times in ice-cold PBS, collected by scraping into PBS supplemented with 1X Complete Protease Inhibitor Cocktail (PIC; Roche), then passaged 3 times through a 18G needle to disrupt cells. Liver from wild-type mice was dissected, blood was removed by perfusion of PBS into the hepatic vein, and the tissue was disrupted in PBS/1X PIC using a Tissue Tearor homogenizer (VWR). HeLa cell and mouse liver extracts were centrifuged at 200,000g for 15 min at 4 ^o^C, and the resulting supernatants were divided into aliquots and stored at -20 ^o^C.

Samples of frozen human cerebellum were homogenized in Lysis Buffer (50 mM Tris-HCl, pH 7.5, 5 mM EDTA, 100 mM NaCl, 0.5% Triton X-100, 10% glycerol, 10 mM K_2_HPO_4_, 0.5% NP-40), supplemented with 1X PIC (Roche), 1 mM sodium vanadate, 0.5% deoxycholate, 0.1 mM PMSF, 20 mM NaF, and 20 mM glycerol 2-phosphate. Tissue homogenates were centrifuged at 13,000g for 15 min and the resulting supernatant was stored in aliquots at -80 °C until further use. Protein concentrations were estimated using a bicinchoninic acid (BCA) assay (Sigma) according to manufacturer’s recommendations.

### Immunoprecipitations

For immunoprecipitations, 10 µg of each anti-IDE mAb or normal mouse IgG as a control was added to tubes containing 320 µg protein extracts supplemented with 0.2 % NP-40, which were incubated with agitation for 2 h at 4 ^o^C. Protein G-agarose beads (Roche) were pre-equilibrated by addition of IP Buffer (PBS, 1X PIC, 0.2% NP-40) 4 times, then 10 µL bead volume was added to each tube, which were incubated with agitation for 1 h at 4 ^o^C. The beads were washed 4 times in IP Buffer, 1 additional time in detergent-free IP Buffer, then the pellets were resuspended in 1X Novex Tris-Glycine SDS PAGE Sample Buffer (Invitrogen) supplemented with 5 mM ß-ME.

### Western Blot Analyses

Soluble protein extracts from cells (30 µg/lane) or immunoprecipitated samples (10 µL/lane) were subjected to SDS-PAGE under reducing conditions using 10% polyacrylamide tris-glycine mini gels (Invitrogen) and transferred to nitrocellulose membranes as described [5]. Membranes were blocked in 5% non-fat milk in tris-buffered saline supplemented with 0.2% Tween-20 (TBS-T). For western blots, membranes were cut into segments, each incubated for 1 h at room temperature with individual anti-IDE mAbs (1 µg/mL). For immunoprecipitations, membranes were incubated for 1 h at room temperature with a rabbit polyclonal antibody raised against IDE (αIDE-1; 1:1000; [6]; generous gift of Dennis Selkoe, Harvard Medical School). Membranes were washed extensively with TBS-T, probed with peroxidase-conjugated anti-rabbit IgG secondary antibodies (1:5000; Vector Labs), and detected by enhanced chemoluminescence using SuperSignal West Pico Substrate (Pierce).

For brain extracts, equal amounts of protein (80 μg/lane) and known amounts of recombinant IDE (5-100 ng or ~68.8-1376.5 pM in 15 μL) were electrophoresed on Criterion^TM^ XT 10% Bis-Tris gels (Bio-Rad), followed by transfer to a polyvinylidene fluoride membrane (MilliPore). IDE levels in brain extracts were assessed in triplicate, and normalized to an internal control comprised of extracts from a single case that was run on all western blots (see *Statistical Analysis* section). MagicMark™ XP molecular weight marker (Invitrogen) was used as a size standard. Membranes were blocked with 0.5% Casein Hammarsten (USB) in PBS and then incubated with mouse mAb against IDE, 2A1 (diluted in 0.5% Casein/PBS 1:300). Anti-mouse antibodies labeled with Alexa Fluor 680 (Invitrogen) diluted 1:20000 in 0.5% Casein/PBS/1%SDS/0.1%Tween20 were applied after washing the blots 5 times with TBST. IR detection was performed using the Odyssey Infrared Imaging System (LI-COR Biosciences). Blots were imaged at 169 μm scan resolution. Quantification was performed with the analysis software provided, according to the manufacturer’s instructions. Glyceraldehyde-3-phosphate dehydrogenase (GAPDH) stained with a rabbit polyclonal anti-GAPDH antibody (1: 50,000; Affinity BioReagents) was used as a loading control.

### Immunocytochemistry

Immortalized hepatocytes from IDE-KO and wild-type mice were grown on glass cover slips (Fisher Scientific), fixed by treatment with 4% formaldehyde in PBS for 30 min at 22 ^o^C, washed with PBS 2 times, and permeabilized by treatment with 0.02% Triton X-100 in PBS. After washing with PBS 2 times, the cover glass was treated with RNase A diluted 1:100 in PBS supplemented with 10% horse serum, and 1% BSA for 30 min at 37 ^o^C, washed 2 times with PBS, then incubated with individual anti-IDE mAbs dissolved in PBS/1% BSA (5 µg/mL) overnight at 4 ^o^C. After 2 washes with PBS, cells were treated with Alexa-488-conjugated anti-mouse IgG (2 µg/mL; Cell Signaling Technology) dissolved in PBS/10% horse serum/1% BSA for 1 h at 22 ^o^C. After 2 washes with PBS, the nuclei of cells were stained by treatment with propidium iodide (Abcam) dissolved in PBS (1 µg/mL) for 10 min at 22 ^o^C. After 2 washes with PBS, cover slips were mounted on slides using PermaFluor™ Aqueous Mounting Medium (Thermo Scientific) according to manufacturer’s recommendations.

Confocal microscopy was conducted using a Zeiss LSM 510 META confocal laser scanning microscope. Conventional fluorescent and bright field microscopy was conducted on a Nikon Eclipse 80i, with a Q imaging 12 bit CCD camera.

### Immunohistochemistry and Immunoflourescent Staining on Human Brain Tissue

6A1 and 6H9 were characterized for both immunohistochemistry (IHC) and immunofluorescence (IF) applications using archived post-mortem, paraffin-embedded human brain tissue. Cases were chosen from the Mayo tissue registry that were considered pathologically normal under routine neuropathological examination with post-mortem intervals of ≤15 h. Hippocampal and cerebellar regions were chosen and tissue sections were cut at ~5 μm, deparaffinized in xylene and run through a dehydrating EtOH gradient. Tissue was pretreated with steam for 30 min for antigen retrieval and washed 3 times in PBS. For IHC staining, endogenous peroxidase activity was quenched by incubating tissue sections in a 3% solution of H_2_O_2_. Slides were run on an automated stainer (Dako Autostainer Plus) using the manufacturer’s 3,3’-diaminobenzidine (DAB)-based autostainer kit (Dako Envision+ System HRP anti-mouse). Sections were incubated overnight in primary antibody (40 µg/mL) at 22 ^o^C for IHC and 4°C for IF labeling. For IF, sections were treated with secondary flourochromes (Alexa Flour 488-conjugated anti-mouse IgG and AlexaFluor 568-conjugated anti-mouse IgG; Molecular Probes) diluted 1:500 in antibody dilution solution (Dako) for 90 min at 22 ^o^C, washed 3 times with PBS. Sections processed for IF were immersed, following primary incubation; in a solution of 1% Sudan black B (1g/100ml of 70% EtoH) for 3 min to block autoflourescent pigment. Sections were then cover slipped in Vector Shield H-1200 mounting media (Vector Laboratories). A no-primary negative control was used for all IF and IHC staining procedures. For IF and IHC microscopy analysis, a Nikon Eclipse 80i, with a Q-imaging 12 bit CCD camera was used.

### Human IDE Sandwich ELISAs

To develop ELISAs for quantitative detection of human IDE, a subset of anti-IDE mAbs was first conjugated with horse radish peroxidase (HRP) using the EZ-Link Plus Activated Peroxidase Kit (Thermo Scientific) according to manufacturer’s recommendations. Nunc-Immuno Microwell 96-well plates (Fisher Scientific) were coated (100 µL/well) with unmodified mAbs diluted in PBS to a range of concentrations (2 – 50 µg/mL), and incubated overnight at 4 ^o^C. The coating solutions were discarded and replaced with Blocking Solution (PBS supplemented with 1% Block Ace (AbD Serotec) and 0.05% NaN_3_; 300 µL/well) and incubated overnight at 4 ^o^C. Plates were washed 2 times with PBS (300µL/well), then recombinant IDE standards (1.7 to 27.2 ng/well or 156 to 2500 pM in 100 uL) diluted in Buffer EC (0.05% CHAPS, 2 mM EDTA, 400 mM NaCl, 0.2% BSA, 0.4% Block Ace, 0.05% NaN_3_ in 20 mM sodium phosphate buffer, pH 7.0) were applied (100 µL/well) and incubated overnight at 4 ^o^C. Plates were washed 2 times with PBS, then HRP-conjugated mAbs diluted 1:1000 in Buffer C (2 mM EDTA, 400 mM NaCl, 1% BSA, 0.002% thimerosal in 20 mM sodium phosphate buffer, pH 7.0) were applied (100 µL/well) and incubated overnight at 4 ^o^C. Plates were washed 3 times with PBS supplemented with 0.05% Tween-20, then developed using the SureBlue TMB (3,3’,5,5’-tetramethylbenzidine) peroxidase substrate (Kirkegaard & Perry Laboratories) according to manufacturer’s recommendations.

For quantification of IDE in human brain extracts, ELISA plates were coated with 6H9 (2 µg/mL) and detected with HRP-conjugated 6A1 (1 µg/mL). Protein extracts from human cerebellum (100 µg/well) were dissolved in Buffer EC and processed as above. Brain extracts from each subject were assessed in triplicate. Brain extracts from 2 subjects were run as internal controls on each of the ELISA plates for normalization as discussed below.

### Statistical Analysis

Absolute IDE levels in human brain extracts from western blots and ELISAs were calculated using standard curves comprised of known amounts of recombinant IDE. Samples showing a coefficient of variation (CV) ≥ 20% or a mean lying outside the range of the standard curve were excluded from the analyses. To correct for inter-experimental variability, data were first normalized to internal control samples included across all experiments, then absolute protein levels were calculated using the mean IDE concentration of the internal control sample(s) across all gels or plates. Linear regression analysis was calculated using StatsDirect v2.5.8. (StatsDirect, Ltd.)

# References

1. Farris W, Leissring MA, Hemming ML, Chang AY, Selkoe DJ: **Alternative splicing of human insulin-degrading enzyme yields a novel isoform with a decreased ability to degrade insulin and amyloid beta-protein.** *Biochemistry* 2005, **44:**6513-6525.

2. Shen Y, Joachimiak A, Rosner MR, Tang W-J: **Structures of human insulin-degrading enzyme reveal a new substrate mechanism.** *Nature* 2006, **443:**870-874.

3. Zhao J, Li L, Leissring MA: **Insulin-degrading enzyme is exported via an unconventional protein secretion pathway.** *Mol Neurodegener* 2009, **4:**4.

4. Leissring MA, Farris W, Wu X, Christodoulou DC, Haigis MC, Guarente L, Selkoe DJ: **Alternative translation initiation generates a novel isoform of insulin-degrading enzyme targeted to mitochondria.** *Biochem J* 2004, **383:**439-446.

5. Leissring MA, Farris W, Chang AY, Walsh DM, Wu X, Sun X, Frosch MP, Selkoe DJ: **Enhanced proteolysis of beta-amyloid in APP transgenic mice prevents plaque formation, secondary pathology, and premature death.** *Neuron* 2003, **40:**1087-1093.

6. Vekrellis K, Ye Z, Qiu WQ, Walsh D, Hartley D, Chesneau V, Rosner MR, Selkoe DJ: **Neurons regulate extracellular levels of amyloid beta-protein via proteolysis by insulin-degrading enzyme.** *J Neurosci* 2000, **20:**1657-1665.
